# Variation and molecular evolution of HmbR, the *Neisseria meningitidis* haemoglobin receptor

**DOI:** 10.1099/mic.0.036475-0

**Published:** 2010-05

**Authors:** Nicholas J. Evans, Odile B. Harrison, Kirsten Clow, Jeremy P. Derrick, Ian M. Feavers, Martin C. J. Maiden

**Affiliations:** 1 Faculty of Life Sciences, University of Manchester, 131 Princess Street, Manchester M1 7DN, UK; 2 National Institute for Biological Standards and Control, South Mimms, Potters Bar, Hertfordshire EN6 3QG, UK; 3 The Department of Zoology, University of Oxford, South Parks Road, Oxford OX1 3PS, UK

## Abstract

Meningococcal disease caused by serogroup B *Neisseria meningitidis* remains an important health problem in many parts of the world, and there are currently no comprehensive vaccines. Poor immunogenicity, combined with immunological identity to human sialic acids, have hindered the development of a serogroup B conjugate vaccine, resulting in the development of alternative vaccine candidates, including many outer-membrane protein (OMP)-based formulations. However, the design of protein-based meningococcal vaccines is complicated by the high level of genetic and antigenic diversity of the meningococcus. Knowledge of the extent and structuring of this diversity can have implications for the use of particular proteins as potential vaccine candidates. With this in mind, the diversity of the meningococcal OMP HmbR was investigated among *N. meningitidis* isolates representative of major hyper-invasive lineages. In common with other meningococcal antigens, the genetic diversity of *hmbR* resulted from a combination of intraspecies horizontal genetic exchange and *de novo* mutation. Furthermore, genealogical analysis showed an association of *hmbR* genes with clonal complexes and the occurrence of two *hmbR* families, A and B. Three variable regions (VR1–VR3), located in loops 2, 3 and 4, were observed with clonal complex structuring of VR types. A minority of codons (3.9 %), located within putative surface-exposed loop regions of a 2D model, were under diversifying selection, indicating regions of the protein likely to be subject to immune attack.

## INTRODUCTION

*Neisseria meningitidis*, a Gram-negative diplococcus, is a major cause of meningitis and septicaemia worldwide (Tzeng & Stephens, 2000). Successful vaccines, based on conjugate-capsular polysaccharides, have been developed against meningococcal serogroups A, C, W-135 and Y (Mitka, 2005); however, poor immunogenicity, combined with the immunochemical similarity of the meningococcal serogroup B polysaccharide to human glycoproteins, have hampered the development of vaccines against serogroup B meningococci (Finne *et al.*, 1983). Outer-membrane proteins (OMPs) are widely regarded as leading candidates in the search for alternative vaccine components, and various OMPs have been proposed as vaccine components, including PorA, PorB, FetA, TbpB, NspA, NadA and fHbp (Black *et al.*, 1986; Jacobsson *et al.*, 2009; Jodar *et al.*, 2002; Madico *et al.*, 2006; Wedege *et al.*, 1998). As most meningococcal OMPs are diverse, inadequate cross-protective immune responses to heterologous strains have been attained by most vaccines produced so far (Tappero *et al.*, 1999; Urwin *et al.*, 2004). In several instances, however, OMP-containing outer membrane vesicle (OMV) vaccines have been effective against the particular epidemic strain from which they were derived (Bjune *et al.*, 1991; Sierra *et al.*, 1991; Thornton *et al.*, 2006).

The haemoglobin receptor protein (HmbR) is a meningococcal OMP which has not been included in vaccine formulations to date. It is a TonB-dependent receptor that binds haemoglobin, extracts the haem and transports it into the periplasm (Perkins-Balding *et al.*, 2003; Stojiljkovic *et al.*, 1995). It has a molecular mass of 89 kDa and, like other pore-forming OMPs and TonB-dependent receptors, it is predicted to adopt a transmembrane *β*-barrel structure consisting of 22 putative transmembrane regions with 11 extracellular loops (Perkins-Balding *et al.*, 2003). Evidence for the importance of the haemoglobin receptor gene (*hmbR*) in meningococcal virulence has been described (Harrison *et al.*, 2009; Stojiljkovic *et al.*, 1995), with expression of the gene controlled at the level of transcription by the Fur repressor, and at the level of translation by slipped-strand mispairing at a polyguanosine (G) tract located within the coding region (Lewis *et al.*, 1999).

The diversity of the *hmbR* gene has been compared for a limited number of meningococcal isolates, one for each of the serogroups A, B and C, as well as for the gene in the related organism *Neisseria gonorrhoeae* (Stojiljkovic *et al.*, 1996). Sequences are highly conserved within the three meningococcal isolates, whilst the gonococcal *hmbR* contains a premature stop codon, leading to the expression of a non-functional protein. The extent of the diversity of the gene in large meningococcal populations is poorly defined to date. Here, a comprehensive analysis of *N. meningitidis hmbR* diversity was undertaken using a panel of meningococcal isolates, which included representatives of the principal hyper-invasive lineages (Maiden *et al.*, 1998). Genealogical analysis identified two *hmbR* families with clonal complex structuring among both. Analysis of selection and recombination on *hmbR* sequences revealed that a number of residues, located in regions corresponding to putative surface-exposed loops, were subject to strong positive selection and recombination.

## METHODS

### *N. meningitidis* strains.

The 107 *N. meningitidis* isolates employed in the evaluation of the multilocus sequence typing (MLST) typing method were investigated (Maiden *et al.*, 1998). The collection, which was assembled to be representative of organisms causing endemic and epidemic disease in the latter part of the 20th century, included several isolates from each of seven recognized hyper-invasive clonal complexes: sequence type (ST)-1, ST-5, ST-4, ST-11, ST-32, ST-8 and ST-41/44. All meningococcal isolates were grown on Mueller–Hinton agar, supplemented with 5 % (v/v) defibrinated sterile horse blood, for 15 h at 37 °C in a 5 % (v/v) CO_2_ atmosphere. DNA was extracted from all strains using an IsoQuick Nucleic Acid Extraction kit (ORCA Research) in accordance with the manufacturer's instructions.

### Nucleotide sequence determination.

The *hmbR* genes were amplified using *Taq* DNA polymerase (AmpliTaq, Perkin–Elmer) with PCR primers PN1, 5′-CCGGAAGGAATGATGCCGCACAGG-3′, and NE3, 5′-TTAAAACTTCCATTCCAGCG-3′. For optimal PCR amplification of the *hmbR* gene, reaction mixes were incubated for 30 cycles; each cycle consisted of 94 °C for 1 min, 60 °C for 2 min and 72 °C for 4 min. PCR products were purified using a PEG precipitation method (Embley, 1991). Sequence termination reactions of the purified PCR products used the BigDye Ready Reaction mix according to the manufacturer's instructions (Applied Biosystems), with the sequencing primers listed in Supplementary Table S1. Sequence termination reaction products were separated and the sequence data collected using an ABI Prism 377 automated DNA sequencer (Applied Biosystems).

### Gene assembly and alignment.

Sequence data were assembled using the staden sequence analysis package, with both strands of DNA sequenced for each strain (Staden, 1996). Initial sequence alignment and manipulation employed seqlab from the GCG package (Womble, 2000). The upstream non-protein-coding region together with the first 23 codons, encoding the signal peptide and the stop codon, were removed to leave only the mature peptide-coding sequence. In addition, genetic diversity arising from the slipped-strand mispairing region encoded by the polyG tract was removed. The sequences were aligned to maintain maximum positional homology of the nucleotide sequence with consideration for codon constraints. A database was established containing *hmbR* sequences obtained in this study (http://neisseria.org/nm/typing/hmbR).

### Analysis of sequence data.

mega version 4 was used to calculate overall *p-*distances of *hmbR* nucleotide sequences as well as the within- and between-family and clonal-complex-associated *p-*distances (Tamura *et al.*, 2007). Genealogical trees were constructed using clonalframe version 1.1 (Didelot & Falush, 2007). Inference was performed in a Bayesian framework and a neutral coalescent model was assumed, based on the hypothesis that the bacteria in the sample came from a constant-sized population in which each bacterium was equally likely to reproduce irrespective of its previous history. In the present study, five independent runs each consisting of 100 000 iterations and 100 000 burn-ins were performed with every hundredth tree sampled. These were then assessed for convergence and combined, after which a 75 % majority rule consensus tree was derived. Annotation was then undertaken by importing the tree into mega version 4.

### Analysis of selection pressure.

The start2 program was used for tests of selection using the ratio of non-synonymous to synonymous nucleotide substitutions (*d_N_*/*d_S_* ratio) (Jolley *et al.*, 2001). A phylogenetic tree representative of the *hmbR* alleles was constructed using the treepuzzle program (Schmidt *et al.*, 2002). The HKY nucleotide substitution model (Hasegawa *et al.*, 1985) and a gamma distribution of rate variation among sites (with eight rate categories and shape parameter *α*) were used for tree reconstruction. Values for transition : transversion ratio, gamma distribution shape parameter (*α*) and base frequencies were estimated by the program from the dataset.

Selection pressures acting on meningococcal *hmbR* were investigated with a maximum-likelihood method (Yang, 1997; Yang *et al.*, 2000). Current recommended models used in this analysis were: M0 (one ratio), M1a (nearly neutral), M2a (positive selection), M7 (beta) and M8 (beta and *ω*) (Wong *et al.*, 2004; Yang *et al.*, 2005). Nested models were compared using a likelihood ratio test (LRT). When parameters for each positive selection model were estimated, a naive empirical Bayesian (NEB) approach was used to infer the most likely category for each codon site (Nielsen & Yang, 1998; Yang, 1997). It has been shown that detecting positively selected sites using NEB under certain conditions can give rise to a large number of false positives (Suzuki & Nei, 2004; Zhang, 2004). These anomalies are due to the failure of the NEB approach to account for maximum-likelihood parameter estimate sampling errors. A subsequent Bayes empirical Bayes (BEB) analysis was used to accommodate uncertainties in maximum-likelihood estimation of parameters in the *ω* distribution using numerical integration (Yang *et al.*, 2005). All the analyses described above were implemented using the codeml program from the paml package version 4b (Yang, 1997; Yang *et al.*, 2005).

Characterization of selection in the presence of recombination was carried out using the omegamap software package (Wilson & McVean, 2006). This method employed a Bayesian approach to parameter estimation that was independent of phylogeny and therefore less likely to falsely identify sites subject to diversifying selection in sequences displaying clear evidence of recombination. The signature of natural selection was detected using the *d*_N_/*d*_S_ ratio and the signature of recombination was detected from the patterns of linkage disequilibrium. In the present study, three runs composed of 100 000 iterations and 100 000 burn-ins each were undertaken, compared to assess convergence and combined. Output from the omegamap runs was used to visualize possible selection acting on the sequence by means of a graph indicating the posterior probability of positive selection along the sequence. The consensus from all runs was also imported into Microsoft Excel and manipulated to generate a graph showing the levels of positive selection acting on each codon (data not shown).

### Generation of an HmbR structural topology model.

A method for *β*-strand prediction employing hidden Markov models and neural networking (Martelli *et al.*, 2002), with a reported residue accuracy of 83 %, was used to produce a structural topology model of HmbR. The results from the prediction were critically inspected by comparison with a protein sequence alignment produced using clustal w (Thompson *et al.*, 1994) of HmbR against other TonB-dependent receptors whose structures have been determined: *Escherichia coli* FhuA, FepA, BtuB and FecA, *Pseudomonas aeruginosa* FptA and *Serratia marcescens* HasR (PDB IDs: 1QFF, 1FEP, 1NQG, 1KMP, 1XKW and 3CSL, respectively). To produce the structure-based sequence alignment, the protein sequences were aligned using clustal w and manually adjusted to reflect structural information provided by Krieg *et al.* (2009). The deduced protein sequence from allele *hmbR01* was randomly chosen for this analysis.

## RESULTS

### Patterns of diversity

PCR *hmbR* amplicons were obtained from 89 of the 107 *N. meningitidis* isolates, resulting in 52 unique sequences ranging from 2295 to 2307 bp, with allele *hmbR01* the most frequent (Table 1 and Supplementary Table S2). The remaining 18 *hmbR*-negative isolates contained *exl2*, *exl3* or *exl4* ORFs and have been described elsewhere (Harrison *et al.*, 2009). The level of sequence diversity was assessed by calculating *p-*distances, revealing the proportion (*p*) of nucleotide sites at which sequences differed. Nucleotide alleles had average *p-*distances varying from 0.012 to 0.116, with a total of 426 polymorphic nucleotide sites. Many of the polymorphisms occurred within the putative surface-exposed loops 2, 3, 4, 5 and 10. The 52 *hmbR* alleles encoded 44 unique deduced amino acid sequences. Among putative HmbR proteins, there were a total of 121 variable residues, including one indel at codon 323 and two simultaneous indel pairs at codons 676–677 and 680–681.

**Fig. 1.**
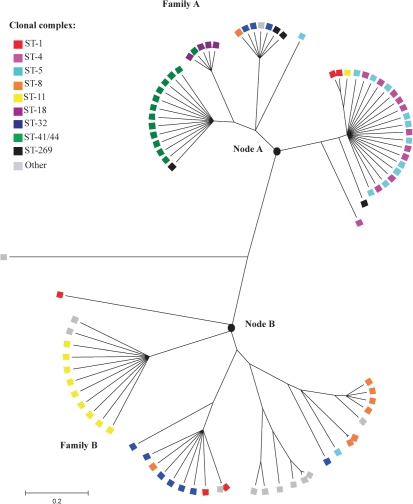
clonalframe analysis of the *hmbR* sequences. Phylogenetic trees were constructed using clonalframe version 1.1 available at http://www2.warwick.ac.uk/fac/sci/statistics/staff/research/didelot/clonalframe/ (Didelot & Falush, 2007). In the present study, over 100 000 iterations and 100 000 burn-ins were performed with every hundredth tree sampled, after which a 75 % majority-rule consensus tree was derived. Annotation was then undertaken by importing the tree into the Molecular Evolutionary Genetics Analysis software package (mega v4.0) (Tamura *et al.*, 2007).

Two families of *hmbR* genes were evident following clonalframe analysis (Fig. 1, Supplementary Table S2). A total of 56 % of sequences belonged to family A and included *hmbR* genes from isolates in clonal complexes ST-4, ST-5, ST-18, ST-41/44 and ST-269, as well as three ST-32, one ST-8 and two ST-1 isolates, whilst 43 % of isolates occurred in family B and included sequences from the clonal complexes ST-11, the remaining ST-32, ST-8 and ST-1 isolates, as well as many other unique STs. One serogroup Z ST-28 isolate contained a hybrid *hmbR* gene. Mean *p-*distances within families A and B were 0.027 and 0.049, respectively, with a mean *p-*distance of 0.076 between both families. The indel observed at codon 323 was predominantly found among HmbR peptides associated with family B, although this was not exclusive, while indels 676–677 and 680–681 were not related to either family. The division of the *hmbR* sequences into two families was apparent in the deduced peptide alignment of loops 2, 3 and 4 beginning at residue 197 through to site 340 (Supplementary Fig. S1). Allelic variants of the deduced peptides from loops 2, 3 and 4 [denoted variable region (VR)1, VR2 and VR3, respectively] were determined, from which a correlation between *hmbR* family and VR combination was observed (Supplementary Table S2), with family A *hmbR* sequences predominantly containing VR types 1, 1, 1 or 2, 1, 2, and family B including 3, 2, 3 or 1, 3, 5 VR combinations. A web-accessible database was established to enable future querying of *hmbR* sequences and submission of new allele sequences (http://neisseria.org/nm/typing/hmbR). Using this database, the VR type of each *hmbR* sequence can also be determined.

Genealogical examination of the *hmbR* genes using clonalframe also provided evidence for antigenic structuring, with *hmbR* sequences from isolates belonging to the same clonal complex tending to cluster together. A total of nine isolates belonging to the ST-41/44 complex contained the *hmbR08* allele and were well conserved (mean *p-*distance=0.001). Five of the isolates belonging to the ST-11 clonal complex shared the *hmbR22* allele and could be found in the same clade (mean *p-*distance=0.011). ST-4 and ST-5 *hmbR* sequences of Serogroup A were clustered, with many harbouring the *hmbR01* allele and with mean *p-*distances of 0.003 and 0.013, respectively. However, the *hmbR* alleles belonging to the ST-1, ST-8 and ST-32 complexes included both families A and B and exhibited more diversity, with mean *p-*distances of 0.059, 0.034 and 0.048, respectively, although some clonal complex structuring was still observed (Fig. 1).

### Evidence of recombination and positive selection

**Fig. 2.**
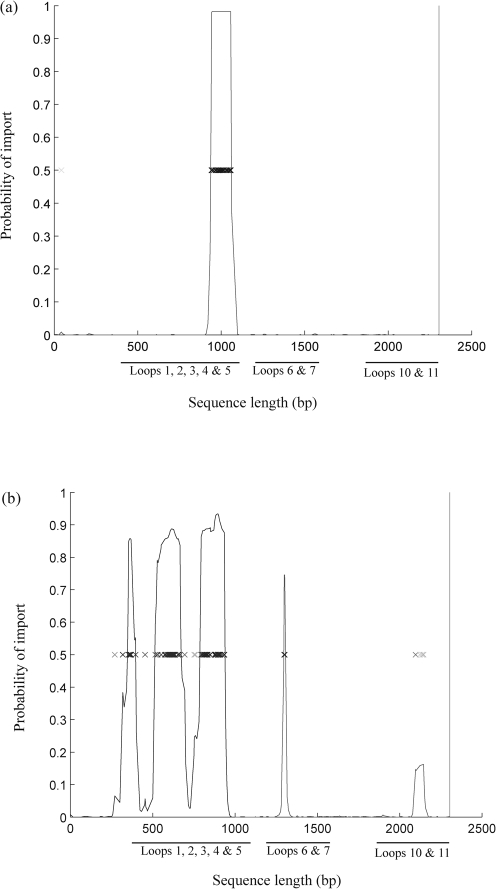
clonalframe representation of *hmbR* recombination events. The nucleotide sequence of *hmbR* genes is represented on the *x* axis, with the grey line indicating at each locus the probability for an import on a scale from 0 (bottom of the *y* axis) to 1 (top of the *y* axis). Each inferred substitution in the graph is represented by a cross, the intensity of which indicates the posterior probability for that substitution. In (a), a single recombination event occurring at node A among family A *hmbR* sequences is represented (Fig. 1). In (b), horizontal genetic exchange at node B and among family B sequences is depicted occurring approximately from bases 300 to 500, 500 to 750 and 750 to 1000. The amino acid location of each loop is represented with additional functional information (Perkins-Balding *et al.*, 2003): loop 1, 484–501; *loop 2, 589–681 haem binding; *loop 3, 775–831 haem binding; *loop 4, 928–1020; *loop 5, 1147–1218; loop 6, 1315–1377 haem utilization; loop 7, 1465–1545 haem utilization; *loop 8, 1627–1764; loop 9, 1870–1905; *loop 10, 1993–2067; and *loop 11, 2161–2274. Asterisks indicate loops containing positively selected sites.

Genealogical analysis indicated very little horizontal genetic exchange between families A and B, although recombination events were apparent among isolates within each family (Fig. 2). Among family A isolates, horizontal genetic exchange was apparent in one short region before and after nucleotide site 1000 (Fig. 2a, node A). Numerous recombination events were observed among family B isolates, particularly in the N-terminal region of the sequence (Fig. 2b, node B).

The *hmbR* gene had an average *d*_N_/*d*_S_ ratio of 0.16, indicating a level of purifying selection against amino acid change. A codon-by-codon examination of *d*_N_ and *d*_S_ ratios (parameter *ω*) using models of codon substitution which differed in the extent to which *ω* varied along sequences was employed to detect evidence of selection at individual codons. Phylogenetic relationships obtained from the sequences were incorporated into the models, resulting in independent comparisons (Yang *et al.*, 2000). Models used were: M0, which assumed a single *ω* parameter for all sites; M1a, which divided codons into conserved sites (*p*_0_), with *ω*_0_ <1 and neutral sites (*p*_1_), with *ω*_1_ set to 1; M2a, which accounted for positive selection through a third category of sites, *p*_2_, with parameter *ω*2 ≥1; and models M7 and M8, which both used a discrete *β* distribution (with 10 categories and described by parameters *p* and *q*) to model *ω* ratios among the sites, although M8, unlike M7, considered an extra class of sites for which *ω* could be >1.

Nested models [differing by extra parameter(s)] were compared using an LRT in which twice the difference in log-likelihood between models was compared with the value obtained under a *χ*^2^ distribution. The degrees of freedom were equal to the difference in the number of parameters between models. When parameters for each positive selection model were estimated, an empirical Bayesian approach was used to infer to which category each codon site belonged. The posterior probability that a site with a particular dataset belonged to category k (with ratio *ω*k) was calculated. The category k that maximized the posterior probability was the most likely category for the site. Positively selected sites (i.e. sites belonging to the third category with *ω* >1) were then identified and BEB was used to correct for uncertainties in maximum-likelihood estimation.

In each comparison, the LRT statistics were significantly (*P*<0.0001) in favour of models that allowed positive selection (M2a and M8) (Table 2). Inference of positive selection using the M2a model and BEB suggested that 14 residues (1.8 %) were under diversifying selection (*ω*2=3.604), whereas 86.8 % of codons were under strong purifying selection (*ω*0=0.008). The M8 model using BEB predicted 30 positively selected sites (3.9 %), including those predicted using BEB for the M2a model. Similar results were obtained using the omegamap program (data not shown), indicating that these sites were not false positives resulting from high levels of recombination occurring in the meningococcus.

### Construction of a structural topology model

**Fig. 3.**
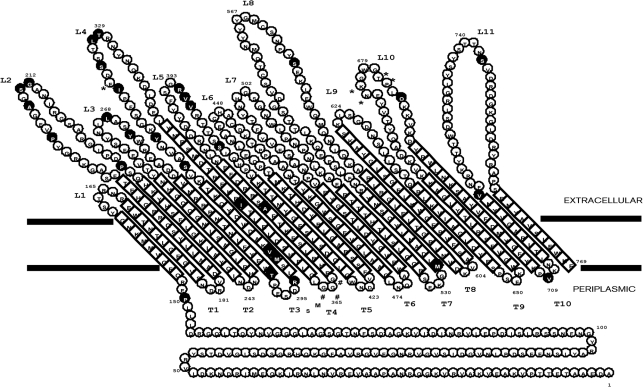
Location of positively selected residues on a structural topology model of HmbR. Positively selected codon sites (black background with white lettering) inferred using the maximum-likelihood method with BEB and the M8 model are identified on a structural topology model of HmbR. Amino acids that correspond to inserted/deleted codons are indicated by asterisks. The glycines encoded by the polyG tract are indicated by hashes.

A hydrophobicity plot was employed to predict *β*-strands for HmbR (Supplementary Fig. S2). By analogy with the crystal structures of other TonB-dependent receptors, HmbR would be expected to adopt a 22-stranded *β*-barrel structure, with an N-terminal ‘cork’ region within the barrel. The presence of a ‘cork’ domain was apparent in the hydrophobicity plot through the absence of transmembrane segments (hydrophobic peaks) before residue 152. The poor signal, short length and singularity of the predicted *β*-strand at positions 561–568 caused it to be allocated to part of a loop region. The signal from residues 325 to 382 was also ambiguous. Visual inspection of sequence alignments against structural homologues was used to predict this region as consisting of two *β*-strands, 341–362 and 377–382. The final topology model for HmbR comprised 22 *β*-strands with 10 smaller periplasmic loops and 11 larger external loops (Fig. 3). The largest loop regions were L8 and L11, consisting of 46 and 38 amino acids, respectively. Extracellular loops L6 and L8 both contained pairs of cysteines (residues 447/450 and 560/570, respectively), which could potentially form disulfide bonds in each putative loop region. To complement the 2D topology model, HmbR was compared with known TonB-dependent siderophore receptors, the cobalamin receptor BtuB and the *S. marcescens* haem receptor HasR, whose crystallographic structures have been determined (Buchanan *et al.*, 1999; Chimento *et al.*, 2003, 2005; Ferguson *et al.*, 1998, 2002; Krieg *et al.*, 2009; Locher *et al.*, 1998). A structural alignment was generated, revealing that all receptors shared the same structure, with each containing 22 *β*-strands, 10 periplasmic loops and 11 surface-exposed loops. The larger extracellular loops 2, 4, 8 and 11 in HmbR were noticeable, and conserved sequence motifs described previously were observed (Supplementary Fig. S3) (Chimento *et al.*, 2005).

Positively selected sites, identified from the BEB and the M8 selection models were mapped onto the HmbR receptor topology model (Fig. 3). The majority (21 out of 30) were situated within predicted extracellular loop regions, with loops 2, 3, 4, 5, 8, 10 and 11 containing positively selected sites. Of the 10 positively selected codons located outside the extracellular loops, seven were situated within the barrel *β*-strands, two on a periplasmic loop and one within the globular N-terminal domain. The glycine residues encoded by the polyG tract were on periplasmic loop T4. A single codon indel occurred on extracellular loop 4 and two codon indel gap pairs on loop 10.

## DISCUSSION

Iron-regulated OMPs are among the numerous potential vaccine candidates present in the cell envelope. This class of proteins includes receptors that are involved in the uptake of iron from transferrin (Legrain *et al.*, 1993), lactoferrin (Prinz *et al.*, 1999), haemoglobin (Stojiljkovic *et al.*, 1995) and hapto-haemoglobin (Lewis *et al.*, 1997). As the concentration of free iron in the human body is insufficient to support microbial growth, the ability to exploit multiple iron sources is believed to play an important role in colonization and dissemination of meningococci in the human body. Additionally, these proteins display conserved functional domains (i.e. the ligand-binding sites) at the cell surface to which the immune response may be directed. Therefore, the iron-limitation-inducible OMPs constitute a group of highly promising vaccine candidates. The vaccine potential of both transferrin and lactoferrin receptors has been evaluated, revealing that both receptors are immunogenic and able to induce bactericidal antibodies, but that the cross-reactivity of these antibodies is limited (Pettersson *et al.*, 2006; Rokbi *et al.*, 1997). The suitability of the HmbR receptor as a vaccine component has not been assessed.

The levels of diversity of *hmbR* (52 alleles encoding 44 unique amino acid sequences) were similar to a number of other vaccine candidate OMPs investigated with this same panel of isolates: PorA (33 alleles encoding 33 peptides); PorB (31 alleles encoding 28 peptides); FetA (33 alleles encoding 31 peptides); and Opa (90 alleles encoding 83 peptides) (Callaghan *et al.*, 2006; Urwin *et al.*, 2004). Furthermore, *hmbR* alleles clustered with clonal complexes in a similar way to other immunogenic OMPs, including the *porA*, *porB*, *fetA* and *opa* genes (Fig. 1, Supplementary Table S2) (Callaghan *et al.*, 2006; Urwin *et al.*, 2004). The presence of two families of *hmbR* gene was reminiscent of the variation observed with meningococcal *porB* (Derrick *et al.*, 1999). PorB proteins can be divided into two classes, class 2 and class 3, a distinction originally based on their size as measured by SDS-PAGE, but PorB2 and PorB3 are also homology groups on the basis of nucleotide and amino acid sequences, as observed currently with HmbR (Derrick *et al.*, 1999; Frasch *et al.*, 1986). Sequencing of *porB* genes has further identified four regions denoted VR1–VR4 located in loops I, V, VI and VII, respectively, enabling *porB* typing in a similar to that of *porA*, which contains two VRs located in surface-exposed loops I (VR1) and IV (VR2) of PorA (Bash *et al.*, 1995; Feavers *et al.*, 1992; McGuinness *et al.*, 1993; Suker *et al.*, 1994; Zapata *et al.*, 1992). Three VRs (VR1–VR3) located in loops 2, 3 and 4, respectively, were also observed in *hmbR* (Supplementary Fig. S1). As with *porA* and *porB*, typing of the *hmbR* VRs revealed that combinations of VR types were found with both *hmbR* families and thus with clonal complexes (Supplementary Table S2) (Sacchi *et al.*, 1998). It has been documented that immune selection by the host can cause populations of meningococci to self-organize into stable collections of strains with non-overlapping repertoires of dominant polymorphic determinants, despite frequent horizontal genetic exchange (Gupta *et al.*, 1996). The non-overlapping association observed among the putative surface-exposed VR peptides identified in HmbR indicates that the immune response directed against these putative epitopes is strong enough to structure the population into discrete combinations of VR types, as evidenced with PorA (Gupta & Maiden, 2001; Gupta *et al.*, 1996).

A structural topology model for the HmbR protein has been described, although it is based on an arbitrary *β*-strand length of 10 residues (Perkins-Balding *et al.*, 2003). Hidden Markov models and neural networking methods were used to predict the *β*-strands of HmbR more accurately (Martelli *et al.*, 2002). As a result, the topology model proposed here, although similar to that previously described, has different-length *β*-strands (Fig. 3). The deduced HmbR peptide was included in a structural alignment containing the *S. marcescens* haemoglobin receptor HasR, as well as the TonB-dependent receptors FhuA, FepA, BtuB, FecA and FptA, whose crystallographic structures have been determined (Buchanan *et al.*, 1999; Chimento *et al.*, 2003; Ferguson *et al.*, 1998, 2002; Krieg *et al.*, 2009; Locher *et al.*, 1998) (Supplementary Fig. S3), revealing that all receptors shared the same structure, with each containing 22 *β*-strands, 10 periplasmic loops and 11 surface-exposed loops. Conserved structure motifs previously described in the N-terminal ‘cork’ domain (also known as ‘plug’ or ‘hatch’) were found as well as the NLFD (also occurring as NLFN or NVFN) motif in loop 11. The cork domain has an essential role in haem uptake, such that combinations of residues from the three apices A, B and C located in the cork domain contribute to ligand binding in these receptors, although individual receptors use different combinations to bind their respective ligand (Krieg *et al.*, 2009). The exact role played by the apices in the cork domain of HmbR has not been determined; however, the presence of the conserved motifs, in addition to the description of HmbR mutants devoid of the cork domain unable to use haemoglobin, is indicative of an essential function (Perkins-Balding *et al.*, 2003). The NPNL conserved motif in loop 7 found in all receptors transporting haem into the periplasm was also apparent, although, in agreement with a previous study, this was not as conserved among the siderophore receptors (Bracken *et al.*, 1999). Histidine residues often serve as axial ligands for haem binding, with two histidine residues in HasR, His-603 in loop7 and His-189 in apex C, conserved among haemophore receptors (Izadi-Pruneyre *et al.*, 2006). HmbR shared one conserved histidine residue (His-500) with HasR (His-603) in loop 7; however, it has previously been shown that mutagenesis of histidine residues in loop 7 does not affect haemoglobin binding in HmbR, indicating that unlike HasR, these residues do not interact with haem (Perkins-Balding *et al.*, 2003).

Functional studies of the HmbR protein have indicated that loops 2 and 3 are necessary for haemoglobin binding, with deletion mutation resulting in 33 and 9 % haemoglobin-binding activity, respectively, compared with the wild-type strain (100 %) (Perkins-Balding *et al.*, 2003). Clonal complex structured combinations of VR1 and VR2, which correspond to loops 2 and 3, were observed (Supplementary Table S2). Thus, despite the fact that the surface-exposed ligand-binding site was under immune selection, as observed by the identification of positively selected residues (Fig. 3), the clonal complex structuring of loops 2 and 3 indicated lineage conservation of the haemoglobin-binding site. The non-overlapping structured combinations of *hmbR* VR peptides including loops 2 and 3 combined with the detection of positively selected residues within these regions is consistent with their being putative epitopes towards which an immune response may be directed.

In contrast, extracellular loops 6 and 7 did not contain residues under strong diversifying selection. These loops are putatively implicated in haemoglobin utilization, with mutation resulting in reduced fitness of the organism (Perkins-Balding *et al.*, 2003). The highly conserved amino acid sequence NPNL identified in haem receptors belonging to bacteria including *Yersinia enterocolitica* and *Haemophilus influenzae* was also observed in loop 7 of the meningococcal HmbR proteins in this study (Bracken *et al.*, 1999). The lack of recombination events evidenced by clonalframe analyses (Fig. 2, nodes A and B) is also indicative of the conserved nature of loops 6 and 7. Positively selected sites were also located in surface-exposed loops 4, 5, 8 and 10, which are predicted to be large extracellular regions and have not been implicated in haemoglobin binding or utilization (Perkins-Balding *et al.*, 2003). Correspondingly, these loops were found to contain numerous polymorphic sites and displayed some of the highest *p-*distances in this study as well as many instances of horizontal genetic exchange (Table 2) (Fig. 2, node B). The putative redundant role of these loops may therefore allow greater diversity. Indeed, VR typing of these regions was not as structured (data not shown).

Although most residues within the transmembrane *β*-strands of HmbR were highly conserved, 10 positively selected sites were located among these structural regions (Fig. 3), although combined with the data obtained using omegamap, individual *d*_N_/*d*_S_ ratios for these sites were low, indicating a lower probability of positive selection. Many of these were found to be connected mutations such that a proline residue at site 150 was invariably associated with a glutamic acid residue at site 151, whereas an aspartic acid residue at site 150 was always linked to another aspartic residue at site 151. Similarly, a methionine residue at site 532 was consistently linked with serine and glutamic acid residues at sites 528 and 529, respectively, whereas a threonine residue at site 532 was accompanied by glycine and aspartic acid residues at sites 528 and 529. According to the topology model, four positively selected sites (positions 291, 296, 299 and 300 in Fig. 3) were located close to one another on opposite sides of a *β*-turn. A methionine residue at position 291 was invariably accompanied by tryptophan, serine and leucine residues at positions 296, 299 and 300, whereas a tryptophan residue at position 291 was accompanied by arginine, methionine and valine residues at positions 296, 299 and 300. Within the limitations of the homology model, the side chains of the two residues may be in contact, suggesting that these are compensatory mutations. Elsewhere, the reasons for positive selection within the *β*-barrel region were more difficult to discern.

Molecular epidemiology has played an important role in the development, implementation and study of meningococcal vaccines. For candidate protein components it is essential to identify the diversity of the protein in suitable bacterial populations, ideally before a vaccine formulation is tested in humans. Thus, although the suitability of the HmbR protein as a vaccine component through the use of functional assays has not been investigated, comprehensive analysis of *N. meningitidis hmbR* diversity provides the first step in revealing the potential of the gene. Data presented here support the conclusion that HmbR is an OMP whose diversity bears clear signatures of a dynamic interplay with the host immune system. A web-accessible database has been created (http://neisseria.org/nm/typing/hmbR), enabling current and future *hmbR* sequences to be queried or deposited whilst also allowing the VR type of each *hmbR* sequence to be determined. It is hoped that as this database grows, it will be constructive in establishing the molecular epidemiology of the *hmbR* gene in *Neisseria* populations.

## Figures and Tables

**Table 1.** Sequence diversity of *hmbR*

**Parameter**	**Value for *hmbR***
Number of alleles	52
Number of isolates of which alleles are representative	89
Length of sequence	2295–2307
Number of variable sites	426
Sequence divergence:	
Mean *p-*distance	0.058
Range	0.04–0.116
*d*_N_/*d*_S_	0.164
Number of amino acid sequences	44
Number of variable sites	121
Mean *p-*distance:	
Loop L1	0.0
Loop L2	0.135
Loop L3	0.095
Loop L4	0.184
Loop L5	0.109
Loop L6	0.051
Loop L7	0.056
Loop L8	0.076
Loop L9	0.011
Loop L10	0.062
Loop L11	0.036

**Table 2.** Likelihood values, selection pressure parameter estimates and positively selected sites of the *hmbR* gene

**Model code**	***p***	**ℓ**	***d*_N_/*d*_S_**	**Estimates of parameters**	**Likelihood test**	***χ*^2^**	***P***	**Positively selected sites†**
M0 (one-ratio)	1	−15 011.4	0.138	*ω*=0.138				
M1a (nearly neutral)	2	−13 676.9	0.137	*p*_0_=0.870 *p*_1_=0.130*ω*_0_=0.007 (*ω*_1_=1)	M0 vs M1a	2669	<0.0001	
M2a (positive selection)	4	−13 573.5	0.221	*p*_0_=0.868 *p*_1_=0.101(*p*_2_=0.031) *ω*_0_=0.008(*ω*_1_=1) *ω*_2_=3.604	M0 vs M2a M1 vs M2a	2875.8206.8	<0.0001<0.0001	150P*, 205P*, 209A*, 211K, 225P*, 268T*, 299M*, 300V*, 312A, 394R*, 396V*, 401S*, 532M*, 709V
M7 (beta)	2	−13 636.1	0.117	*p=*0.011 *q=*0.069				
M8 (beta and *ω*)	4	−13 541.1	0.212	*p*_0_=0.973 (*p*_1_=0.027)*p*=0.008 *q*=0.048*ω*=3.592	M7 vs M8	190	<0.0001	150P*, 205P*, 209A*, 211K*, 212G*, 225P*, 268T*, 271S, 291T, 296R*, 299M*, 300V*, 312A*, 316Y, 322I*, 325S, 328L*, 329T, 352I, 376A, 394R*, 395V*, 396V*, 401S*, 532M*, 575S, 684Q, 709V*, 721V*, 743S

†Positively selected sites identified using BEB. *Represents above 99 % confidence level, whilst other sites listed are above 95 % confidence level.

## Abbreviations

- BEB, Bayes empirical Bayes
- LRT, likelihood ratio test
- OMP, outer-membrane protein
- ST, sequence type
- VR, variable region
